# Analysis of Different Facets of the Rule of 10 for Cleft Lip Repair for Their Application in the Current Era

**DOI:** 10.7759/cureus.53832

**Published:** 2024-02-08

**Authors:** Sneha Pendem, Raparthi Bhuvan Chandra, Kathiravan Selvarasu, Murugesan Krishnan, Muthusekhar M.R., Preethi J

**Affiliations:** 1 Oral and Maxillofacial Surgery, Saveetha Institute of Medical and Technical Sciences, Saveetha Dental College and Hospitals, Chennai, IND; 2 Oral Surgery, Saveetha Institute of Medical and Technical Sciences, Saveetha Dental College and Hospitals, Chennai, IND; 3 Anaesthesiology, Saveetha Institute of Medical and Technical Sciences, Saveetha Dental College and Hospitals, Chennai, IND

**Keywords:** hemoglobin, pediatric surgery, innovative strategies, novel cleft lip repair, cheiloplasty, rule of 10

## Abstract

Objective: To evaluate the relevance of the "rule of 10" as a deciding factor preoperatively for patients undergoing cleft lip repair in the Indian sub-continent.

Design: A questionnaire survey was conducted.

Setting: All tertiary cleft care centers in the Indian subcontinent participated in an online questionnaire survey with anesthetic and surgical professionals.

Main outcome: The primary goal of this survey was to determine the relevance and applicability of various aspects of the rule of 10 as a preoperative guideline for determining the timing of cleft lip repair in ASA I infants. The survey also aids in understanding the systemic factors that need to be prioritized and factors that are no longer of primary relevance in defining the timeline to undertake cleft lip repair in infants in the current era.

Results: Surgeons and anesthetists from 31 tertiary cleft centers in India responded to the questionnaire. Specifically, 64.5% do not apply the “rule of 10” for deciding the timing of cleft lip repair, and 77% of the centers reported that cleft lip repair can be taken up in infants with hemoglobin levels in the range of 9-10 g/dL and an average weight of 4.5 kg. The average blood loss in unilateral lip repair ranged between 5 and 10 mL and 10 and 40 mL in children with bilateral lip repair. Three to six months was the average age at which cleft lip repair was undertaken at most of the centers in India.

Conclusion: The rule of 10 is not considered a gold standard by most of the centers in India, and the decision-making was based on the overall physiological status of the patients, the experience of the surgeon, and the anesthetic and post-operative care facilities available at the center.

## Introduction

The timing of cleft lip repair has been often debated in surgical society, considering the challenges associated with peri-operative care in infants. The "rule of 10" formulated by Wilhelmsen et al. in 1966 was the first published guideline that defined the timing of cleft lip repair [1]. This was later modified by Millard (1976) and is now used all over the world [2-3]. As per the rule of 10, a cleft lip can be repaired in infants at 10 weeks of age with hemoglobin levels of 10 gm%, an average weight of 10 pounds, and a total count of leucocytes of 10,000 cells/cc. This was based on the high perioperative mortality that was evidenced in that era with minimal perioperative facilities.

On the contrary, recent advances in pediatric and neonatal anesthesiology have led to the development of advanced intubation techniques and ventilation facilities, enabling the anesthetic team to handle difficult airways proficiently in pediatric patients [4]. Apart from this, advances in the fields of pharmacology and surgical armamentarium make neonatal surgery possible, with the earliest intervention being the ex-utero intrapartum treatment (EXIT) procedure that is performed intrapartum. Thus, the rule of 10 may not be a stringent rule that needs to be applied for screening patients undergoing cleft lip repair.

The surgeons in India often face dicey situations as children do not satisfy the rule of 10 due to maternal and infant malnourishment. In these situations, it is difficult to make the decision on the timing of surgery in these infants needing cleft lip repair. Delaying the surgery may be counterproductive as the widening of the cleft is reported to be associated with poorer outcomes [5]. Considering the aforesaid, it is essential to evaluate the basis on which the rule of 10 was formulated, its implications, and the relevance of the individual facets of the rule of 10 in the current era of advanced medical sciences in the Indian sub-continent. The aim of the current survey was to establish the relevance of the “rule of 10” and its different facets as a guideline for deciding the timing of repair in infants undergoing cheiloplasty in the current era.

## Materials and methods

A questionnaire survey was conducted to evaluate the validity of the rule of 10 and its applicability as a cutoff preoperative guideline in patient selection for cheiloplasty.

The questionnaire was designed to effectively capture the data pertaining to the application of the rule of 10 in clinical practice. The relevance of the current questionnaire, the format of the questions, and their relevance in clinical practice were assessed and validated by an expert panel of senior surgeons in the field of cleft and craniofacial surgery. The prototype questionnaire was circulated amongst 14 postgraduate resident doctors and five faculty as a part of the pilot study. The test-retest method of evaluation was conducted to assess the internal consistency and validation. After the validation of the questionnaire, it was circulated amongst operational cleft and cranial centers in India.

The questionnaire primarily focused on gaining knowledge of the surgical and anesthetic fraternities and the applicability of different facets of the rule of 10 in the Indian scenario. Considering that the guidelines of the rule of 10 are based on the difficult airway that matures with age and the physiologic response to blood loss, the questionnaire was constructed to assess the volume of blood loss; complications associated with blood loss; the impact of low hemoglobin on bleeding, healing, and oxygen carrying capacity; the role of body weight in pre-anesthesia preparation; and difficulties or complications encountered with pediatric intubation (Table 1). All the questions were closed-ended to arrive at a definite consensus. The responses received were tabulated and assessed to evaluate the relevance of the different facets of the rule of 10 in the Indian scenario.

**Table 1:** Questionnaire on the rule of 10

QUESTION NO.	QUESTIONS	ANSWERS
1	What is the average blood loss in unilateral cleft lip repair in patients aged between 6 months to 1 year (personal experience)?	(a) 10-20 mL, (b) 20-40 mL (c), 40-60 mL, (d) Above 60 mL
2	What is the average blood loss in bilateral cleft lip repair in patients aged between 6 months to 1 year (as per personal experience)?	(a) 10-20 mL, (b) 20-40 mL, (c) 40-60 mL, (d) Above 60 mL
3	What is the average hemoglobin level of patients reporting cleft lip to your centers for the repair of cleft lip?	(a) Less than 8 gm/dL, (b) 8-9 gm/dL, (c) 9-11 gm/dL, (d) Above 12 mg/dL
4	Have you ever been required to transfuse blood post-cleft lip repair?	(a) Yes, (b) No
5	Do you apply the ‘Rule of 10’ as a standard for case selection in cleft lip repair?	(a) Yes, (b) No
6	Does delay in the surgery of cleft lip impact the long-term outcomes of cleft lip repair?	(a) Yes, (b) No
7	What is the minimum Hb% and weight of the child at your centres for unilateral cleft lip repair?	Answer
8	What is the minimum Hb% and weight of the child at your centres for bilateral cleft lip repair?	Answer
9	How do you proceed with patients presenting with cleft lip with pre-operative lower levels of haemoglobin between 8-10 gm%?	(a) Delay in surgery with nutritional supplements, (b) Proceed with surgery with nutritional supplements, (c) Proceed with the procedure with blood loss-reducing agents (haemostatic agents), (d) Others
10	What complications have you recorded when operating on a child with less than 9 gm% of Hb (surgical and aesthetic)?	a) Increased bleeding, b) Poor healing, c) Intraoperative desaturation, d) None
11	What all preoperative and intraoperative anaesthetic measures are taken in your centres to reduce bleeding in patients with haemoglobin between 8 and 10 gm%?	a) Judicious use of cautery, b) Systemic tranexamic agents, c) Mechanical pressure, d) Nothing specific
12	Age at which repair of cleft lip is undertaken at your centre.	a) < 3 months, b) 3-6 months, c) More than 6 months
13	Your comments on the “Rule of 10” for cleft lip repair.	a) Gold standard, b) Not relevant in the current era, c) Not a standard that always needs to be adhered to

## Results

The questionnaire was forwarded to 40 operational tertiary cleft centers in India with high caseloads. A total of 31 responses were received. Five centers could not be reached, and four centers were non-operational and so were excluded.

**Figure 1:**
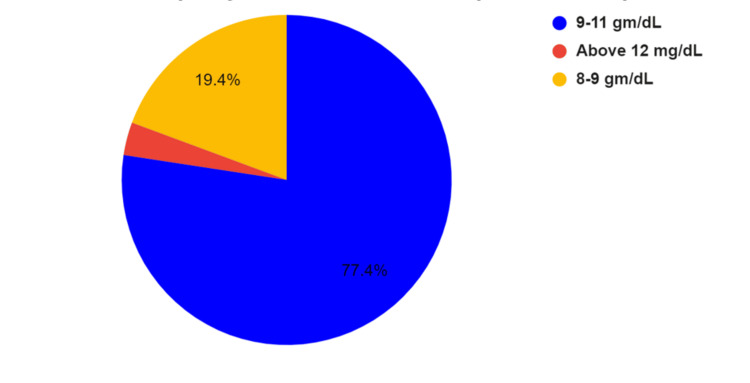
Hemoglobin levels in infants reporting for cleft lip repair

Seventy-six percent of the surgeons stated that the average hemoglobin of the infants reporting to their center ranged between 9 and 11 gm%, with 20% reporting it to be between 8 and 10 gm%, as shown in Figure 1.

**Figure 2:**
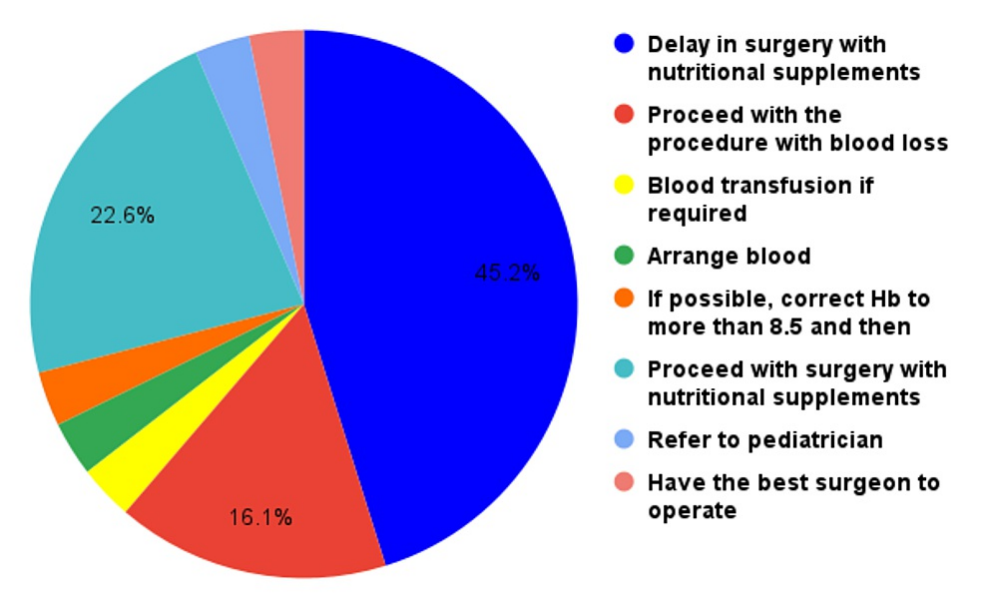
Approach to children with low hemoglobin levels

Most surgeons prefer to operate on children with unilateral cleft lips up to 8 gm% and 4.5 kg weight and bilateral cases with hemoglobin levels up to 9 gm% and 5-6 kg weight. The approach for the children presenting with cleft lips with pre-operative lower levels of hemoglobin between 8 and 10 gm% is depicted in Figure 2.

Additionally, 43.3% preferred to delay the surgical treatment for the patients presenting with hemoglobin levels between 8 and 10 gm%, whereas the rest preferred to proceed with surgery with post-operative nutritional supplementation of iron.

**Figure 3:**
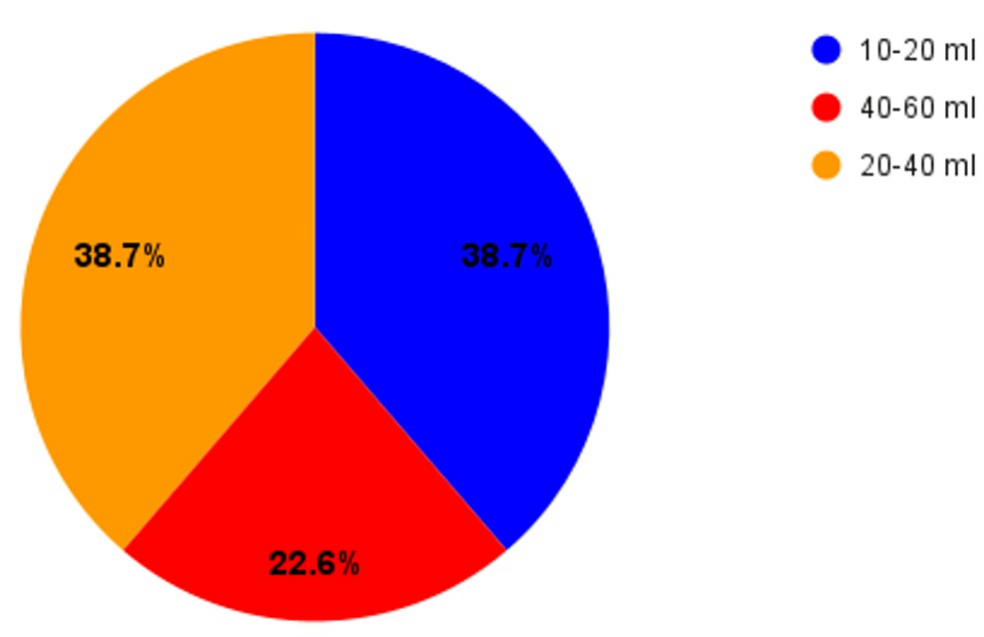
Average volume of blood loss in unilateral cleft lip repair

The average blood loss encountered in unilateral lip repair was stated to be 10-20 mL. Nasoalveolar molding (NAM) was performed only in 20% of the centers, while the rest (80%) of the centers did not undergo NAM due to the lack of resources. There was no significant difference in the volume of blood loss among NAM and non-NAM cases. The blood loss in bilateral cleft lip cases of patients aged between six months to one year was recorded as 10-20 mL by 40% of the surgeons 20-40 mL by 36.7% and 40-60 mL by 23.3%, as shown in Figure 3. None of the surgeons reported the need to transfuse the blood in patients with unilateral or bilateral cleft lip patients.

On average, 64.5% of the surgeons do not consider the application of the “rule of 10” as a guideline for the selection of cases in cleft lip repair, and 56.7% of the surgeons denied long-term ill effects of delay in surgery.

There were no anesthetic/post-surgical complications in children who violated the rule of 10. Various peri-operative and intra-operative measures have been taken by both surgeons as well as anesthetists while operating on children violating the rule of 10, including judicious use of diathermy, local vasoconstrictors, administration of hypotensive anesthesia, and use of hemostatic agents such as tranexamic acid.

## Discussion

Pediatric surgery is frequently difficult due to anatomical and physiological factors, such as difficult airways, low cardiopulmonary reserve, low circulatory volume, and body weight [6,7]. These are the primary reasons that the surgeon decides to postpone surgical interventions until early infancy. The high incidence of perioperative complications in early surgical intervention has led to the inception of the rule of 10 put forward by Wilhelmsen et al. in 1966 [1,8].

Neonatal physiology is extremely delicate, with a continuous transition from the postpartum phase to infancy taking place. To avoid perioperative mortality, it is critical to maintain this physiological balance during the perioperative period. The primary source of concern is the heart's low hemodynamic reserve and profound vagal tone, which make it vulnerable to common perioperative complications of cardiorespiratory arrest. Anatomically small airways with a narrow subglottic region, extremely sensitive and hyperactive airway reflexes, and extremely sensitive and hyperactive airway reflexes predispose to peri-operative airway obstruction, resulting in respiratory distress.

The infant's immature hepato-renal system is characterized by a gradual transition of fetal hemoglobin to adult hemoglobin as a result of the transition from placental-dependent oxygenation to pulmonary ventilation, which contributes to transient polycythemia and complicates neonatal/infant anesthesiology.

Apart from the aforementioned, complete reliance on a milk-based diet compromises fetal hemoglobin levels, predisposing them to a state of physiological anemia during the first six to nine months of life. All these factors need to be assessed while planning surgical procedures for infants.

High perioperative mortality secondary to the aforesaid factors has been reported in the past, which has led to the inception of the rule of 10. However, understanding the role of these factors in perioperative care in correlation with current medical advances can provide better insight for deciding the relevance of these facets and the rule of 10 for practice in the current era.

Role of hemoglobin levels

Physiologically, the body is prone to various changes in early infancy. One of the foremost changes is the conversion of placental-dependent oxygen circulation to pulmonary ventilation. This exposes the tissue to a relatively high oxygen tension that stimulates the change over to the adult hemoglobin (Hb A1, A2) from the fetal hemoglobin (Hb F). This change is often a slow progression and takes place over the first 8-10 weeks of birth, and the phase is associated with a slow decline in the hemoglobin concentration that settles to a nadir of 10 mg+/- 2 gm% over time [6,9]. Apart from this, an exclusively milk-based diet in infants with low iron supplements also does not favor an increase in hemoglobin levels. These factors contribute to hemoglobin levels of 8-10 g/dL in the first few months of life, which are similar to the findings reported in our study. Hemoglobin levels are of concern during the intra-operative phase to maintain tissue oxygenation during the phase of apnea during intubation. Hence, it is essential to maintain adequate hemoglobin levels to facilitate the same. Apart from these orthopedic studies, adequate oxygen concentration is essential for tissue healing, and tissue hypoxia can compromise the process of wound healing. However, these effects on wound healing were evident only when hemoglobin levels were as low as <6 g/mL.

However, low hemoglobin levels did not interfere with the decision to undergo cleft repair in most of the centers, and no adverse effects, including excessive bleeding (secondary to hemodilution), delayed healing, or intraoperative desaturations, were reported. This is supported by the studies of Liderkemp et al. [7], who stated that critical hemoglobin values are defined as the levels of hemoglobin that are needed to achieve tissue oxygenation based on the oxygen demands of the tissue [6]. Based on their studies, they stated that the critical hemoglobin level in infants is 6 g/dL and is greater in infants associated with low cardiopulmonary reserves and leukemias [10-12].

Role of weight

The average birth weight of children in the Indian scenario ranges between 3 and 3.5 kg. Postpartum, the first week of infancy, is associated with a decrease in body weight that is followed by a gradual increase at a rate of 500-700 g per week. However, in clinical scenarios, children with facial clefts often remain undernourished due to maternal malnourishment, feeding difficulties, and recurrent upper respiratory infections that can have a catabolic effect [3]. Additional fortified supplements are often necessary for these infants to enhance their overall growth and development. This has led to the inception of the rule of 10 as a baseline physiological guide to assess the child’s physiological readiness for the repair of a cleft lip. As per the rule of 10, the infant needs to be 10 pounds (4.5 kg) at the time of cleft lip repair. Body weight is an important factor that has been favored by most of the surgeons in the survey, as it defines the nutritional status of the patient and is indirect evidence of the physiological readiness of the body for surgery.

Apart from its role in determining the nutritional status of the patient, body weight is also an indirect predictor of the blood volume, thus defining the allowable blood loss, which is an important predictor of surgical outcome.

Though the rule appeared to be preferred by a lot of surgeons in the past, currently it is not considered a standard norm in most of the centers in India. Recent evidence from the Indian scenario has revealed that low hemoglobin levels of up to 8 g/dL with a low weight of up to 4 kg are adequate for the correction of cleft lip repair [2]. Assessment of anesthetic, intraoperative, or postoperative healing complications did not show any significant correlation with either the hemoglobin levels or the body weight up to the aforesaid range. Similar findings were reported in a few studies reporting that none of the facets of the rule of 10, except for the body weight at the time of surgery, seemed to have a predictive prognostic value for complications in children undergoing cleft lip repair [10].

In the sequel to the aforesaid discussion, the current research aims at gaining evidence pertaining to the applicability of the rule of 10 in the Indian scenario. From the results of the study, it is evident that the average hemoglobin of the children presenting for surgery at various centers ranged between 8 and 10 g, with the body weight ranging between 4 and 5 kg. The average blood loss for a unilateral lip repair ranged between 10 and 20 mL, which contributed to 5% of the total blood volume in the infant with a weight ranging between 4 and 5 kg, and the average blood loss in a bilateral lip ranged between 20 and 40 mL, which constituted 5-10% of the total blood volume in the child with a weight ranging between 4 and 5 kg. This change in blood volume did not affect the anesthetic/intraoperative course or the post-operative course, which questioned the validity of hemoglobin as an important facet of the rule of 10 [11].

Low hemoglobin may be an anesthetic risk in patients with associated congenital cyanotic heart disorders or in patients with congenital ventilation-perfusion mismatch issues, in which the hemoglobin levels need to be greater than the baseline to meet the required oxygen demand and compensate for compromised cardiopulmonary status [12-15].

From a surgical perspective, delaying the surgery leads to the widening of the width of the cleft, prompting the use of NAM for a longer duration. Lack of this may be associated with the widening of the cleft, which can have a detrimental effect on the aesthetic outcomes. However, earlier intervention before three months could be challenging considering the anteriorly placed laryngeal inlet and narrow subglottic region. Considering the aforementioned, early surgical intervention immediately after NAM appears to be a good time for surgical repair of the cleft lip.

Most of the surgeons from the Indian sub-continent preferred operating the cleft lip at the earliest when the average weight is between 4 and 5 kg and hemoglobin is 8-10 g/dL. Local hemostatic measures with pressure tamponade over the superior labial artery and the use of electrocautery minimize blood loss and minimize the incidence of post-operative complications [16,17].

Delayed healing in patients with low hemoglobin (<8 g/mL) and a low weight of 4.5 kg was reported by two centers. This is probably due to the overall malnutrition caused by micronutrient deficiency.

Limitations

The current study is a questionnaire study that indirectly emphasizes the lack of support for the application of the rule of 10 in the current era and provides evidence for the use of body weight and age as crucial factors in determining the timing of cheiloplasty in ASA I infants. Though few studies have provided evidence for the same, large-volume multicenter trials are essential in order to revisit and redefine the guidelines for timing of cheiloplasty.

## Conclusions

To conclude, the rule of 10 was a good guideline for the assessment of patients' cleft lip repair. However, it should not be considered a gold standard for defining the timing of cheiloplasty. Patient weight at the time of surgery has taken precedence over the other facets of the rule of 10 to assess the readiness of the physiological status of the patient to undergo the surgical repair. The experience of the surgeon and a good anesthetic facility are important factors that will play a major role in decision-making. The continued evaluation and validation processes are essential for the surgical fraternity to reassess the application of the rule of 10 considering the continued advances in the medical field.
